# Abdominoplasty and rectus diastasis repair-a plastic surgeon’s perspective

**DOI:** 10.1007/s10029-025-03460-2

**Published:** 2025-11-08

**Authors:** Sarah N. Bishop

**Affiliations:** Department of Plastic and Reconstructive Surgery, Cleveland Clinic, 9500 Euclid Ave, A-60, Cleveland, OH USA

**Keywords:** Rectus diastasis, Abdominoplasty, Body contouring, Open rectus diastasis repair, Inter-rectus distance, Abdominal wall laxity, Musculoaponeurotic laxity, Rectus plication, Umbilical transposition

## Abstract

Rectus diastasis repair can be approached in many different ways from robotic to open. However, a large part of the transformation a plastic surgeon can offer a patient who is seeking a diastasis repair is to combine the rectus diastasis repair with body contouring procedures. Patients with excess soft tissues, lax skin or lipodystrophy will benefit from a concomitant body contouring procedure, most commonly in some form of abdominoplasty. Minimally invasive options can be offered; however, patient selection is crucial for the optimal and expected outcome. For patients with a small amount of excess skin, liposuction or other-energy based procedures can be offered to enhance results. Patients should be properly educated on what surgery they are getting and the expected results that can be obtained.

**Supplementary Information:**

The online version contains supplementary material available at 10.1007/s10029-025-03460-2.

## Introduction

Rectus diastasis is defined by the separation of the rectus abdominis muscles in the midline and a subsequent widening of the linea alba which extends from the xiphoid to the pubis. There are many classification systems developed and differences in opinion on what exactly represents a diastasis from a normal width of the linea alba. However, a linea alba greater than 2 cm at a point 3 cm above the umbilicus is generally accepted as pathologic [1–3]. Women typically present with diastasis most marked at the umbilical level as a result of pregnancy whereas men typically present with supraumbilical diastasis from increased intraabdominal pressure from increased visceral fat [4]. Minor rectus diastasis will be typically less than 3 centimeters [5].

Rectus diastasis can be disconcerting at least and even painful and lead to loss of abdominal core strength at its worst [1]. Rectus diastasis is typically approached very differently by plastic surgeons compared to general surgeons. The different approach will often stem from the patient’s biggest concerns whether that be functional or aesthetic. The rectus diastasis repair in a plastic surgeons hands is often approached and taught as an aesthetic problem and therefore the approach will be to maximize shape, contour and scar placement. When rectus diastasis is repaired for primarily aesthetic purposes it is typically combined with an abdominoplasty. Rectus fascia plication will standardly be offered to aesthetic patients even in the face of minimal rectus diastasis.

Minimally invasive surgery has exponentially increased in all surgeries. Rectus muscle plication is no exception to this. SCOLA (Subcutaneous Onlay Laparoscopic Approach) and robotic techniques seek to be the minimally invasive answer to open rectus plication and in certain patient populations can be a good answer [6]. The best candidates for minimally invasive repairs are those patients with isolated rectus diastasis without excess skin or subcutaneous tissue or those that do not care to address the excess.

## Surgical techniques

### Open rectus diastasis repair with concomitant abdominoplasty

Open rectus diastasis repair can be performed with or without abdominoplasty but is most typically performed with abdominoplasty when performed by a plastic surgeon. General Surgeons may approach an open rectus diastasis repair without a concomitant abdominoplasty.

Plastic surgeons use their knowledge of anatomy and more specifically blood supply to manipulate tissues. Understanding vascular supply to the tissues is paramount. It is critical that one understands what blood supply can be sacrificed and what blood supply must be maintained for a certain segment of tissue to live.

Huger described the vascular zones for the abdominal wall and described how the tissues are successfully perfused after an abdominoplasty [7]. Zone 1 perfuses the central abdomen from xiphoid to pubis and is bounded laterally by the rectus abdominis muscles; it is supplied by the arcade formed by the superior and inferior epigastric vessels. Zone 2 perfuses the lower abdomen-connecting a transverse line across the Anterior Superior Iliac Spines (ASIS) and is supplied by the superficial epigastric, superficial circumflex iliac, and inferior epigastric arteries. Zone 3 perfuses the lateral abdomen and flanks and is supplied by the segmental intercostal, subcostal, and lumbar arteries. When elevating an abdominal flap one must ensure that there is one of these vascular zones still intact for the tissues to receive adequate blood supply for survival.

Taylor and Palmer further elucidated the angiosomes and Saint-Cyr the perforasomes to describe in detail the cutaneous blood supply all over the body [8, 9]. Regardless, of how the blood supply is described one needs to be acutely aware of the blood supply and take care to preserve blood supply whenever possible. Perforators encountered often do not need to be sacrificed during surgery.

Abdominoplasty has many different techniques and refinements but the traditional approach is to raise abdominal flaps to the level of the bilateral costal margin and xiphoid, perform rectus fascia plication, translocate the umbilicus and possibly some degree of liposuction as well. In this approach rectus fascia plication is easily tackled as the rectus abdominus muscles are easily in view and accessible. By combining rectus abdominus fascia repair with body contouring procedures one can achieve a true transformation. For many patients the body contouring aspects with excision of excess soft tissue and skin are the most powerful and transformative aspects of an abdominoplasty.

Panniculectomy should be distinguished from abdominoplasty although they share some commonalities. A panniculectomy is considered a functional surgery and may be covered by insurance. For patients with large a overhanging panniculus that causes intertrigo, pain and difficulties ambulating a panniculectomy may be offered [10]. Panniculectomy traditionally avoids undermining tissues, translocation of the umbilicus and rectus plication and is not typically considered a cosmetic operation.

Preoperatively the patient will be examined paying attention to any ventral hernias, abdominal scars, striae, and presence of large amount of intraabdominal fat. Rectus diastasis can be palpated and measured on exam by lying the patient down and having the patient contract their abdominal wall. Imaging can be obtained for concern for hernia formation but is not typically necessary for isolated and uncomplicated rectus diastasis. However, if imaging is performed the inter-rectal distance can be measured and discussed with the patient.

If an asymptomatic umbilical hernia is present it can addressed at the same time as the abdominoplasty; however, this will potentially increase the rate of umbilical necrosis. One can also choose to stage the umbilical hernia repair. During the standard abdominoplasty the umbilical skin will be circumferentially transected (sacrificing the subdermal plexus) so the only blood supply will be through the stalk (from possible branches from deep inferior epigastric vessels and the median umbilical ligament [11]. Concomitant repairs must take into account the remaining blood supply and can be performed laparoscopically or through a small mini-laparotomy incision to avoid any transection to the stalk [12].

Abdominal scars are important to note as these can affect the tissue perfusion of the abdominal flap raised in an abdominoplasty. The subcostal scar is a known concern that can cause tissue necrosis. The traditional teaching is to avoid undermining this scar. Care should be taken to preserve any perforators encountered in this area [13].

Smoking is often considered a contraindication for elective plastic surgery and has well documented and established increased risks for postoperative complications in abdominoplasty and other plastic surgery procedures [14]. Tobacco smoke and nicotine have a multitude of mechanisms that lead to impaired wound healing, decreased oxygen utilization, vasoconstriction, promotion of thrombogenesis and abnormal blood cell function [15–21]. Smoking shows increased risks for wound healing complications, flap necrosis, wound dehiscence, and surgical site infection in abdominoplasty [22–27]. Furthermore, there is the concept of choke vessels which are pre-existing vessels that arborize and connect vascular territories. When perfusion is compromised in surgery we often rely on the choke vessels to dilate and improve the oxygenation to the ischemic tissues [28]. Smoking will further seek to vasoconstrict and offset the body’s natural ability to improve the tissues ischemic state [14].

Particular attention should be given to massive weight loss patients to ensure that they are not anemic or have any nutrient aberrations. Patients that have undergone bariatric surgery are prone to nutrient deficiencies and iron-deficiency anemia [29–31].

The standard approach to an uncomplicated rectus diastasis repair proceeds with a patient under general anesthesia and with complete muscle paralysis. Typically, a lower transverse incision just above the pubis within a skin crease is made along with a circum-umbilical incision. The abdominal flap consisting of the skin and fat overlying the anterior fascia is raised to the bilateral costal margins and xiphoid. The borders of the paired rectus muscles are fully exposed and palpated. Electrocautery can be used to stimulate the muscle to determine the borders. The muscles will then be plicated by invaginating the diastasis inward with a vertical and elliptical closure from the xiphoid to the umbilicus and then a second vertical ellipse from the umbilicus to the pubis. Many will perform a double layer sutured closure with the second layer imbricating the first. Care is taken to not strangulate the umbilicus to avoid necrosis. There is no consensus on the standard suture closure technique whether that be with running, figure-of-8 or interrupted sutures or even absorbable versus non-absorbable sutures. However, a double layered closure is associated with fewer complication rates [1].

**Fig. 1:**
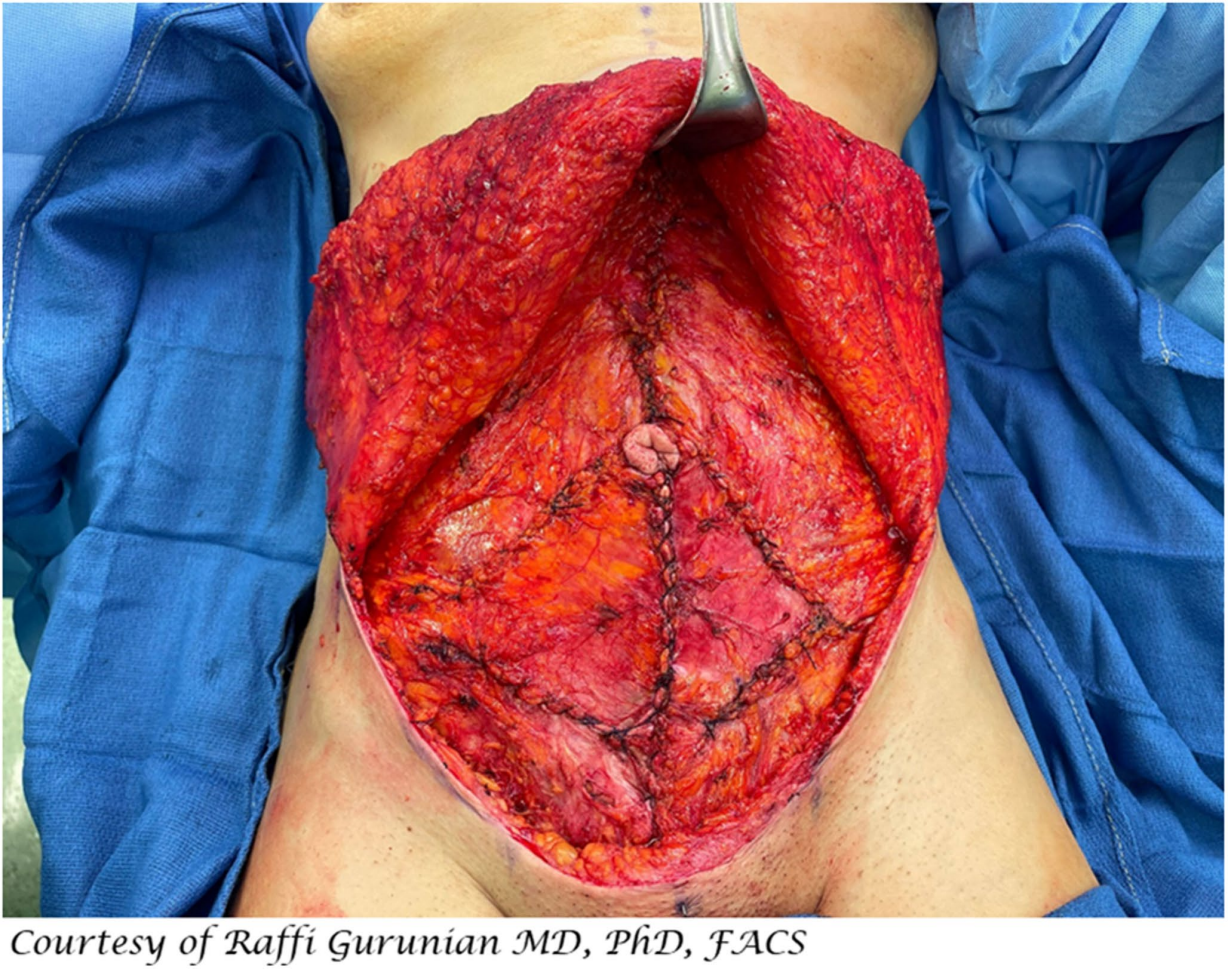
Intraoperative view of open rectus fascia plication showing plication in the vertical and oblique dimensions.

With the open approach one has the most freedom to perform extensive repair of abdominal wall. The degree and the direction of the laxity of the abdominal wall will determine how one should plicate the anterior fascia. Although, fascial plication will usually begin with midline vertical plication, one can also plicate horizontally and obliquely to achieve the desired tautness of the abdominal wall (Fig. 1).

After the plication is complete the patient will be sat up to increase the amount of excess lower abdominal tissue to be excised and offload tension on the closure. The umbilicus can be tagged with a suture for easier retrieval. The umbilical translocation will proceed with insetting of the umbilicus into a new location on the abdominal flap approximately at the level of the ASIS. For patients with a high riding umbilicus and minimal supraumbilical excess skin, floating of the umbilicus can be considered [32]. In this technique no skin incisions are made around the umbilicus and instead the umbilical stalk is transected during raising of the abdominal flap. One should always be mindful of any umbilical hernias. The umbilical base is repaired primarily and after excision of the excess lower abdominal tissue the umbilicus will be re-inset to a new and slightly lower position on the abdominal wall.

**Fig. 2:**
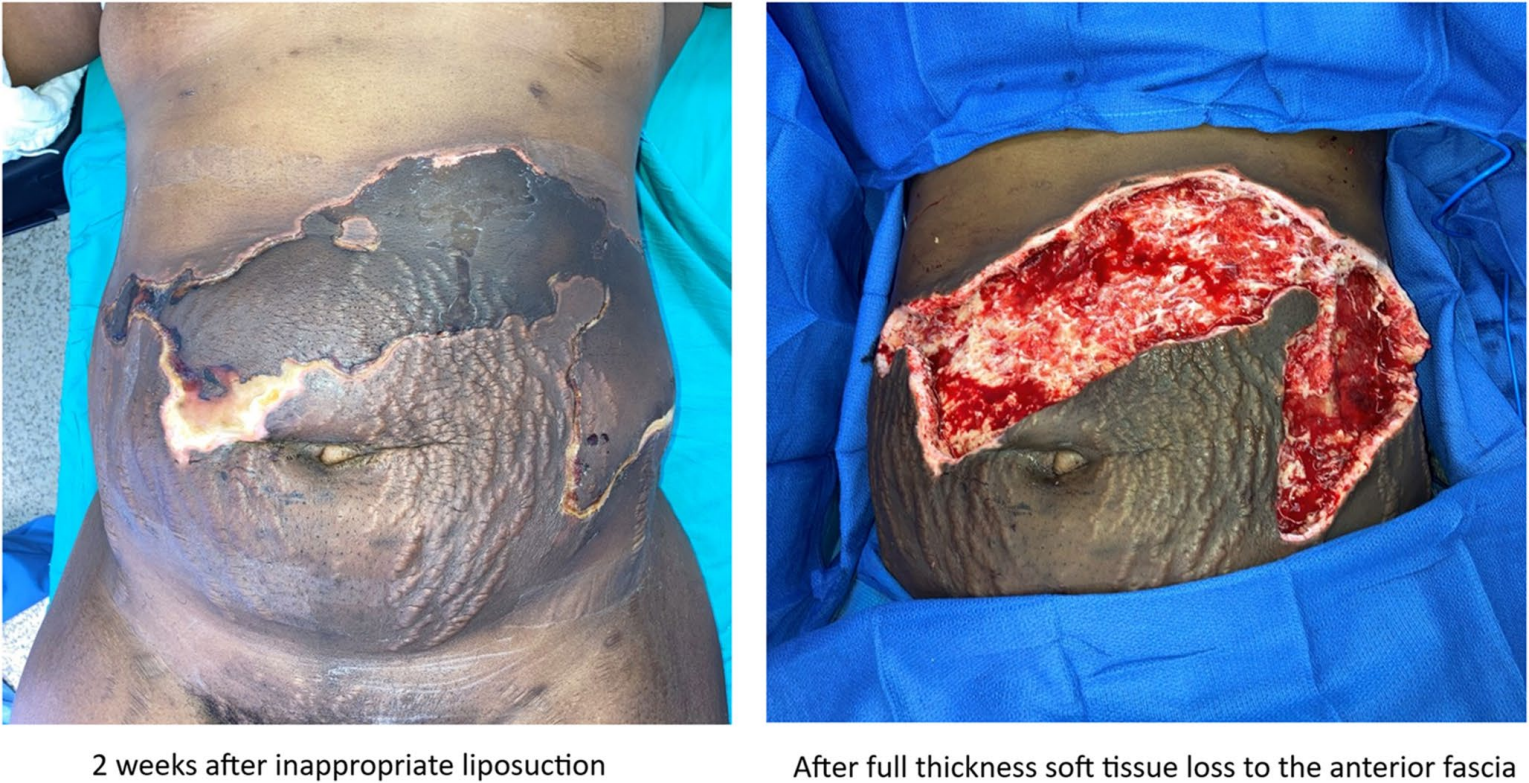
This patient was an unfortunate circumstance of medical tourism. She had loose skin and striae and was therefore not a good candidate for liposuction. In order for liposuction to be safe and effective the overlying tissues need to be healthy enough to retract. Unfortunately, this patient had extensive damage to the abdominal wall soft tissues which required full thickness debridement of the soft tissues all the way to the abdominal wall.

Liposuction is often offered to patients with proper skin tone and necessity. Typically, one avoids liposuction in areas that have been undermined. One should avoid liposuction in tissues that are damaged and with significant striae (Fig. 2). Although, many surgeons will avoid epigastric liposuction to limit abdominal flap necrosis, if perforators are spared than liposuction in this undermined tissue can be safely performed [33].

Seroma is the most common complication. Most surgeons will leave drains but other techniques include progressive tension sutures (to close the dead space and offload tension on the inset) and use of fibrin or other surgical adhesives [34]. Some advocate leaving Scarpa’s down to prevent seroma formation; however, if this layer is thick it can make the rectus plication more difficult [35].

Venous Thromboembolism (VTE) is an unfortunate complication of body contouring procedures and so risk assessment should be obtained and chemoprophylaxis given as necessary [36]. Unfortunately, abdominoplasties have shown the highest risk for VTE of all plastic surgery procedures [37]. There has been a reported 1.2% incidence of Deep Venous Thrombosis (DVT) and 0.8% Pulmonary Embolism (PE) in elective abdominoplasty patients [38]. Efforts to ameliorate VTE risks include both preoperative and postoperative approaches. Preoperatively, one should ascertain a good history and focus on risk factors for perioperative VTE: age greater than 40 years, personal or family history of VTE (which can signal inherited or acquired thrombophilia), smoking, cancer, hormone replacement therapy, estrogen-containing oral contraceptives, selective estrogen receptor modulators, obesity and immobility [37]. Efforts should be taken to improve or address preoperative VTE risks such as quitting smoking, holding hormonal therapies and improving BMI. Intraoperatively, all patients should have Intermittent Pneumatic Compression (IPC) devices and post-operatively early ambulation is essential [39–42]. Many advocate the use of perioperative chemoprophylaxis but there is no true consensus on this yet and is typically up to the surgeon’s discretion [36].

Patients should ambulate as soon possible but may need to walk in a slightly hunched over manner to avoid tension on the closure for a couple of weeks. Patients should sleep in a semi-fowlers or beach chair position to also offload tension. Postoperatively patient will be followed to assess proper wound healing, removal of drains and possible management of seromas or other complications.

There are many varying approaches to abdominoplasty with different incision patterns, different amounts of undermining and liposuction. All approaches should take into account blood supply and the quality of the tissue.

## Open rectus diastasis repair with retrorectus mesh

For patients with very wide diastasis (> 5 cm) the fascial system may be so attenuated that it will not safely hold a sutured repair. For these patients a retrorectus mesh repair can be considered. A retrorectus space is created and a biologic or synthetic mesh, as per the surgeon preference, is placed [43]. An overlay mesh can be placed but generally some kind of underlay mesh will be preferred for optimal mesh performance.

Open rectus diastasis repair with retrorectus mesh can be performed by either a plastic surgeon or general surgeon as long as the surgeon is comfortable and trained. At our institution most patients with a large inter-rectal distance would likely be referred to a Hernia specialist first with plastics as a referral at the discretion of the Hernia surgeon.

## Minimally invasive

### Endoscopic/Laparoscopic

For patients with good quality skin and elasticity one can consider endoscopic lipoabdominoplasty where liposuction is performed first followed by endoscopic or laparoscopic rectus plication. Vila-Rovira developed Extensive Liposuction with Minimal Abdominoplasty (ELMA) particularly for patients with BMI 30–35. In this technique extensive and circumferential liposuction is performed followed by minimal undermining to perform endoscopic rectus plication and umbilical translocation. Finally, excess tissue is removed from a lower and typically shorter transverse scar than traditionally performed [44].

SubCutaneous Onlay Endoscopic Approach (SCOLA) has been described for both rectus diastasis repair and midline ventral hernias. There are several different names to describe this technique but all seek to minimize skin incisions through minimally invasive techniques [6, 45–47].

Laparoscopic ports can be placed through the incisions made for liposuction to offset the number of scars. Typically three trocars are placed, carbon dioxide insufflation is obtained and a supra-aponeurotic space is created to expose the rectus muscles for plication. A suprapubic port will be placed for the camera and bilateral lateral working ports are placed just above the ASIS. Since the umbilical skin is intact than the stalk can be transected and umbilical hernias repaired. The stalk its typically transected to properly visualize the rectus plication but will need to be re-inset to the abdominal wall. Plication can be sutured in the standard fashion. Drains are typically placed to avoid seroma formation [48].

## Robotic

Robotic surgery is on a logarithmic growth curve and many surgeons are developing this niche for which to draw in patients. Current robotic techniques will proceed similarly as the laparoscopic surgeries [49, 50]. The literature is currently sparse in regards to robotic rectus plication but this should change as more and more patients seek out minimally invasive techniques, be they good candidates or not.

For all minimally invasive techniques patients need to be appropriate candidates. If patients have too much subcutaneous fat than plication will noticeably draw and bunch up the tissues unpleasantly. It is important that the tissues drape appropriately to avoid this. This bunched up deformity does not necessarily improve with time as one would hope. Patient selection is critical when it comes to deciding who is the best candidate for minimally invasive options. Patients with increased subcutaneous tissues or loose or redundant tissues will benefit from a concomitant abdominoplasty to simultaneously address these issues. Furthermore, if a patient already has a Pfannenstiel incision than this scar is already present and can be used as a starting point for an abdominoplasty. A patient with prior scars will have less benefit from a minimally invasive approach. However, for patients with good skin quality, with minimal need for body contouring adjuncts or for those who truly do not care about abdominal wall cosmesis than minimally invasive techniques can be offered.

Minimally invasive techniques can be added to endoscopic/laparoscopic rectus diastasis repair such as liposuction, use of energy-devices to tighten the skin and excision of excess tissue too. However, if a large incision is used to resect the excess skin the perceived benefits of the minimally invasive approach are likely diminished.

It is important to note that there are many surgeon websites that advertise robotic techniques for rectus plication and many with beautiful results. However, these can be somewhat misleading as most often these techniques are combined with some other adjunctive techniques such as subcutaneous radiofrequency ablation, liposuction, and/or excision of redundant skin. As long as these treatments are done with the appropriate training there is nothing inherently wrong with this but the patient and other surgeons performing rectus diastasis repair need to understand the true limitations to the laparoscopic or robotic techniques without these adjuncts.

## Discussion

The European Hernia Society Guidelines on the surgical management of rectus diastasis (without concomitant hernia) show that there are no differences when evaluating the surgical treatment with mesh or no mesh and with open versus laparoscopic approaches [51]. Consistently, within the literature there are no differences when evaluating open versus more minimally invasive techniques [52–54]. When there is a concomitant hernia present with rectus diastasis than a mesh repair is recommended by the European and Americas Hernia Society; however, there is still no preferred approach whether that be open or minimally invasive [55]. Fascial plication when combined with ventral hernia repair has also shown to improve Quality of Life and decrease seroma [56, 57].

Patients getting abdominoplasty may have minimal rectus diastasis and small (< 1–2 cm) umbilical hernias and these patients can be candidates for concomitant primary repair of both the rectus diastasis and umbilical hernia [58, 59]. When laparoscopic approach are utilized for the umbilical hernia repair these tend to utilize mesh whereas open approaches use both primary and mesh techniques depending on the size of the hernia [12, 60]. Similarly, when treating other small non-umbilical ventral hernias that lie within the midline, mesh can be avoided if the anterior rectus fascia is felt to be strong enough and the hernia small enough to hold the repair [61].

When evaluating the preferred approach for rectus diastasis repair we need to establish who the patient population is and what are their preferences. Rectus diastasis is a known complication following pregnancy. At 12 months postpartum 33% of women will continue to have persistent rectus diastasis [62]. Women that have had more than one pregnancy are at highest risk. 2% of primiparous women have rectus diastasis compared with 52% of multiparous women [63]. However, there are minimal studies in men addressing rectus diastasis [64].

Pregnancy not only causes rectus diastasis but also significant skin and soft tissue changes. 90% of women will get striae or stretch marks during pregnancy [65]. Striae represent dermal damage and are typically permanent. Larger striae with associated loose skin should alert the surgeon to skin that is at risk for necrosis with surgical procedures [66]. Additionally, loose skin with striae will have permanent changes in the collagen and elastic systems and thus the skin will not retract appropriately with time [67].

**Fig. 3:**
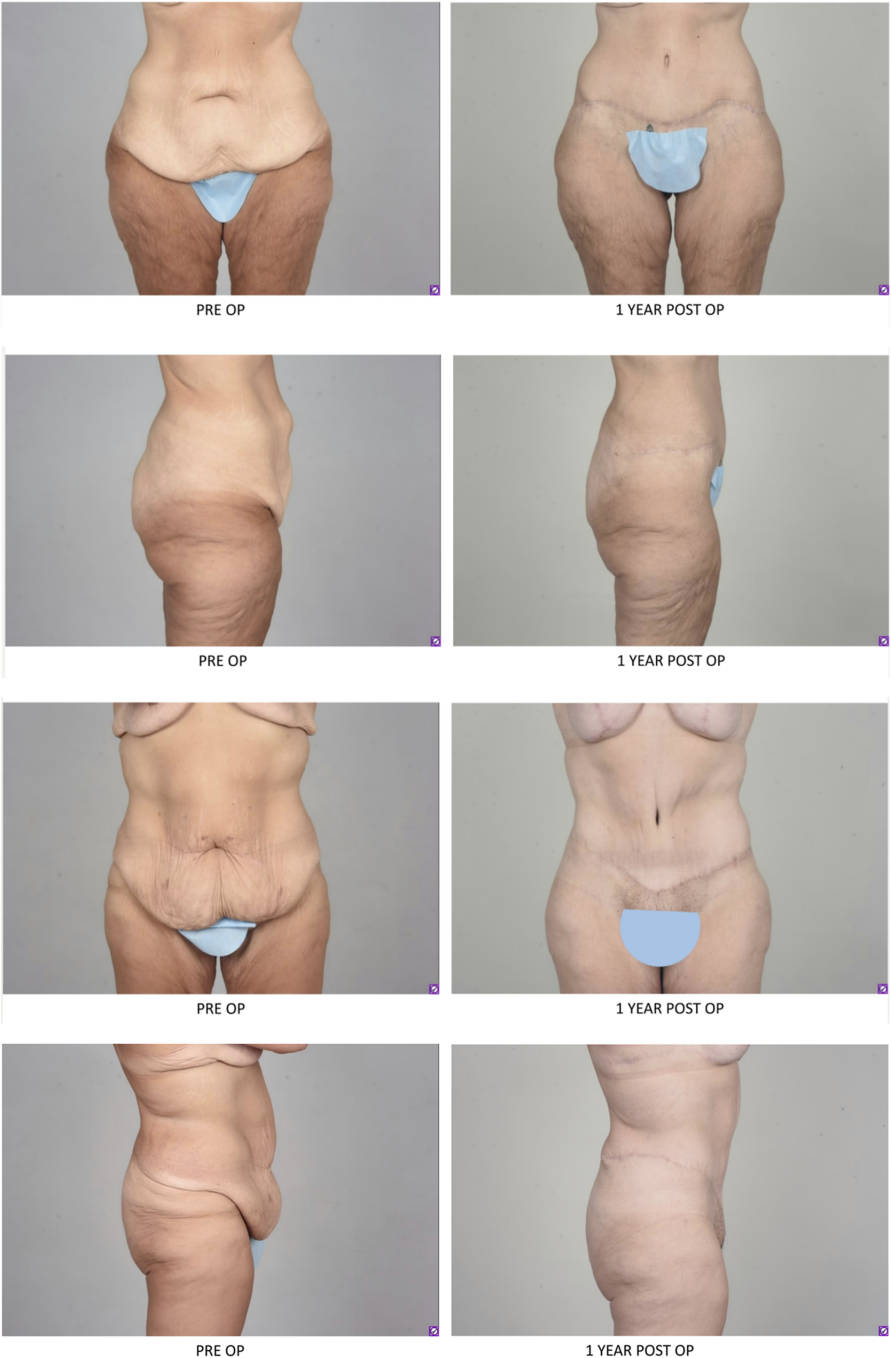
Patients with massive weight loss present with rectus diastasis and excess loose skin. These patients had abdominoplasty (rectus diastasis repair, umbilical translocation and direct lipectomy). These patients did not have liposuction as there was not excess lipodystrophy and the quality of the skin was not amenable to liposuction.

Patients with massive weight loss will also present with many of the same postpartum issues with permanent loose sagging skin and striae (Fig. 3). Massive weight loss leads to loose skin all over ones figure. Body contouring procedures help address these concerns and lead to improved quality of life [68]. Massive weight loss patients will often have excess looseness in both the vertical and horizontal dimensions for which a fleur-de-lis pattern can be applied. In this approach the shape of a fleur-de-lis is drawn on the patient which enables resection of both the vertical and horizontal dimensions, a resulting upside-down T with a circumferential scar around the umbilicus will result [69]. It is important in this technique to avoid wide undermining of tissues that are not resected.

**Fig. 4:**
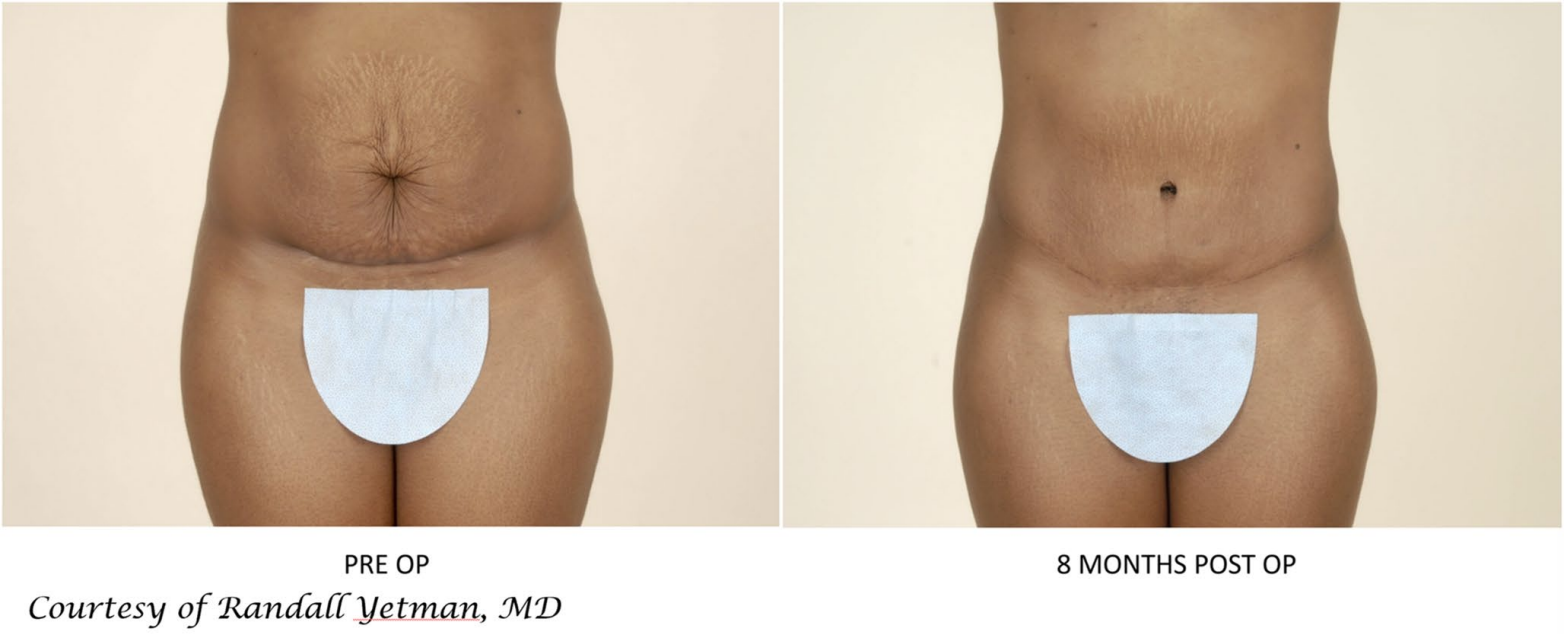
Patient has concomitant rectus diastasis and pregnancy related skin changes (striae, loose skin and Cesarean section scar) that are best addressed by abdominoplasty. Her abdominoplasty proceeded through her prior Pfannenstiel incision.

Although demographics are changing with time, most patients presenting to a cosmetic practice are women. Furthermore, 74% of patients presenting to a cosmetic practice are not only women but mothers [70]. These women presenting to a plastic surgeon most certainly will want not only the rectus diastasis repaired but the excess skin and striae addressed as best as possible. Surgical procedures that only address the rectus diastasis will leave these patients unsatisfied or worse at risk for skin necrosis from minimally invasive procedures that do not excise the damaged skin further damaged from undermining [66]. Finally, 1/3 of women giving birth will do so by Cesarean and will thus have an associated scar [71]. An abdominoplasty can often proceed through this scar and maybe even improve the appearance (Fig. 4).

**Fig. 5:**
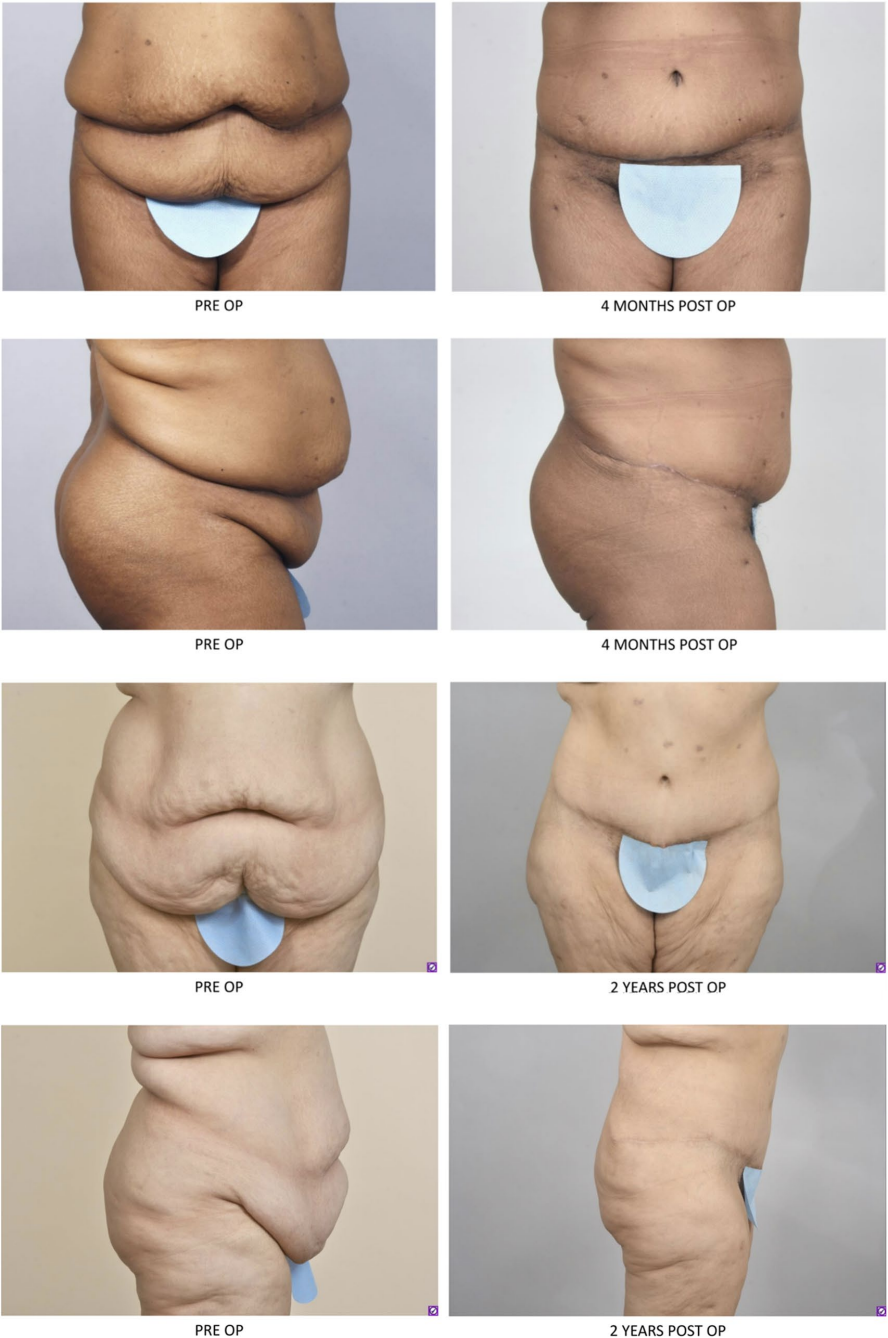
Patients with excess skin and lipodystrophy that benefited from abdominoplasty (flank liposuction, rectus muscle plication, umbilical translocation and direct lipectomy)

Finally, as we assess the patient population with rectus diastasis we should consider the obesity epidemic. 31% of adults are overweight and 19% are obese [72]. Women have higher rates of subcutaneous fat distribution compared to men who often carry more visceral fat [73]. Subcutaneous fat is amenable to liposuction and direct lipectomy (Fig. 5). Traditionally, plastic surgeons have had BMI < 30 for consideration for abdominoplasty. Although, patients with higher BMIs will be at higher risk of surgical complications more surgeons are safely offering abdominoplasties in the obese population [74]. However, patients with large amounts of visceral fat will benefit from weight loss prior to body contouring procedures as rectus plication will be limited and will lead to poor results and outcomes.

The best patients for minimally invasive techniques are those with truly isolated rectus diastasis or those who do not wish to address any soft tissue concerns. Patients that fit these categories are a diminishing patient population. Perhaps the best patient for a minimally invasive technique is the bodybuilder with isolated rectus diastasis. Other patients that are good candidates are those that truly do not care about addressing soft tissue concerns and place a value on minimally invasive techniques and less about aesthetic concerns [54]. When comparing abdominoplasty and minimally invasive techniques there are no differences in: seromas, surgical site infections or hematomas [47, 75–81]. Furthermore, there is no difference in pain, improvement of stress incontinence, or readmission rates [76, 77, 82–84]. Most studies show no difference in regards to rectus diastasis recurrence with the exception of the Robotic TransAbdominal Retromuscular Rectus Diastasis (r-TARRD) repair which had an increased rectus diastasis recurrence with 4 out of 45 patients effected [75]. Minimally invasive surgeries have typically been associated with decreased hospital stay, pain and recovery. However, when comparing abdominoplasty to minimally invasive techniques there is no true metric to prove that minimally invasive techniques enhance recovery except for minimizing the scar [47, 78, 81].

**Fig. 6:**
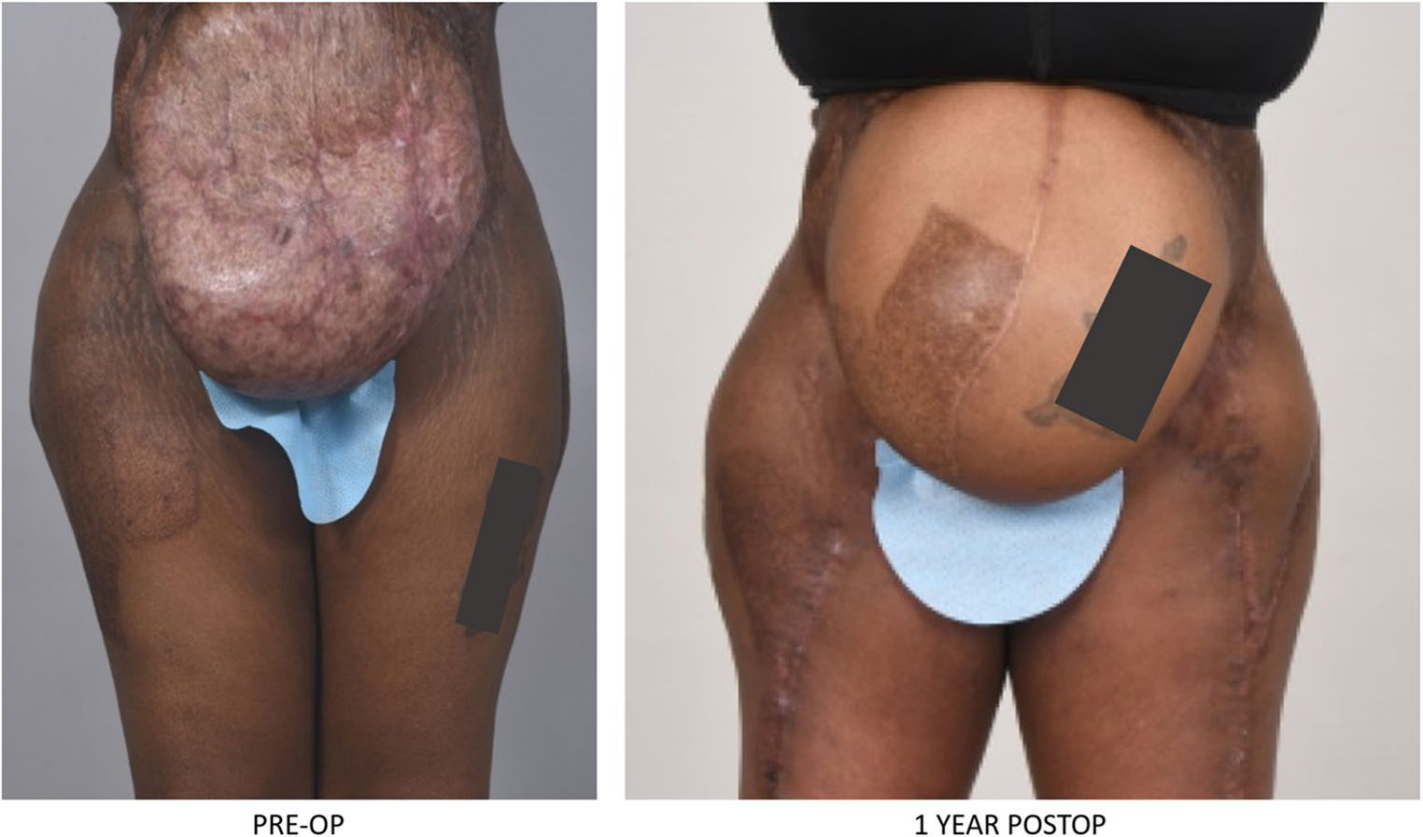
Patient who had devastating liposuction catastrophe at an outside institution with multiple abdominal injuries for which she had multiple surgeries with almost near debridement of the entire abdominal wall. She ultimately was managed with open abdomen and skin graft on bowel. Subsequently, she was referred to our institution and had extensive abdominal wall reconstruction with Transversus Abdominis Release (TAR) and mesh by Dr Michael J Rosen and bilateral neurotized Anterolateral (ALT) with split Vastus lateralis flaps by the author.

Many surgeons will promote minimally invasive techniques for rectus diastasis repair but optimal results are often combined with skin excisions, liposuction and/or adjunctive skin tightening methods with energy-based surgical tools [85]. It is important that patients needs be addressed appropriately but more important that this is done safely. It is imperative that surgeons offering adjunctive techniques have the appropriate knowledge and training to perform these techniques. Skin excision, liposuction and energy-based devices can have devastating complications in the wrong hands (Fig. 6). Plastic surgery, perhaps more than any other specialty, can have unqualified individuals offering surgical treatments to unsuspecting patients [86, 87].

## Conclusion

Rectus diastasis has many options for repair from open to minimally invasive techniques. The type of repair offered should take the patients wants and soft tissue excess into account. If minimally invasive options are pursued than these need to be offered to patients without soft tissue excess or those who prioritize a minimally invasive option over removal of excess tissue. For those patients with a small amount of soft tissue excess minimally invasive options can be used but combined with some combination of: small removal of excess skin, liposuction or energy-based tightening procedures [47, 78–81]. 

## Supplementary Information

Below is the link to the electronic supplementary material.

Supplementary Material 1
